# Coconut Shell Aggregate and Coir Fiber in Cement Concrete: A Review of Mechanical Performance, Durability, and Sustainability Under Functional Equivalency

**DOI:** 10.3390/polym18111383

**Published:** 2026-06-02

**Authors:** Mohammed Mutnbak

**Affiliations:** Civil and Architectural Engineering Department, College of Engineering and Computer Science, Jazan University, Jazan 82817, Saudi Arabia; mmutanbak@jazanu.edu.sa

**Keywords:** coconut shell aggregate, coir fiber, agro-waste, palm oil fuel ash, supplementary cementitious materials, lightweight concrete, durability, interfacial transition zone, functional equivalency, life-cycle assessment

## Abstract

Agricultural waste materials can serve as functional constituents in cement-based composites through three pathways: (i) organic bio-aggregates that lower density and alter thermal behavior, (ii) lignocellulosic fibers that control cracking and improve post-cracking resistance, and (iii) agro-ash supplementary cementitious materials (SCMs) that densify pore structure and reduce permeability when ash quality and curing are controlled. This review draws on 98 papers, with coconut shell aggregate and coir/coconut fibers as the core focus; agro-ash SCMs (notably palm oil fuel ash, POFA, and rice husk ash, RHA) enter where they clarify mechanisms or inform hybrid design. Rather than cataloging compressive-strength data, the synthesis is organized around controllable process inputs (feedstock conditioning, mix design, curing) and the interface-governed mechanisms that determine performance: interfacial transition zone (ITZ) character and pore connectivity. In coconut shell systems, density reductions come at a cost: elastic modulus drops and moisture sensitivity rises unless shell conditioning, particle packing, and matrix refinement are managed. In fiber systems, gains in toughness and residual capacity are bounded by mixing workability and by the long-term stability of the fiber–matrix bond under alkaline and wet–dry exposure. A mix must first meet strength, serviceability, and transport requirements before its embodied impact is compared with conventional alternatives. The contribution is to reframe these systems around controllable processing and interface mechanisms instead of tabulated strength values; preparation, treatment, and characterization data are consolidated into bounded design windows, an explicit core versus supporting evidence convention is applied, and sustainability is judged under functional equivalency rather than per-volume carbon.

## 1. Introduction

Cement-based materials underpin most modern construction, valued for their scalability and low cost. Yet the sector is under increasing pressure to curb its environmental footprint, particularly the carbon intensity of clinker production and the resource demands of large-scale aggregate extraction and transport [1,2,3].

Two practical strategies dominate the path toward lower impact: reducing clinker intensity through SCM substitution or binder redesign, and reducing the material intensity of structures through lower density, optimized cross-sections, or improved durability that extends service life [1,3,4].

Agro-waste materials can close a material loop, but only when they play genuine functional roles in composite systems rather than acting as inert fillers. Whether this works in practice depends on whether the resulting mixes remain mechanically adequate, durable, and constructable for their intended exposure class [1,2,3].

Coconut processing generates large volumes of residues. In the cementitious literature, coconut shell has emerged as the leading candidate for organic lightweight aggregate, while coir and coconut fibers are widely investigated as crack-bridging reinforcements. A parallel body of work explores agro-ashes, including POFA and RHA, as SCMs that reduce clinker demand and refine pore structure [5,6,7,8].

Global coconut production is about 60 to 62 million tons per year [9] (around 60.5 Mt in 2021 [10]). Coconut shell makes up roughly 15.6% of the whole fruit, so on the order of 9.7 million tons of shell are produced each year [9], with coir generated in similar agro-waste quantities [7]; disposing of this large volume of shell residue has become a recognized problem [9]. Converting even part of it into structural-grade material is therefore both a waste-management and a decarbonization opportunity, which is the motivation for this review.

A bibliometric review of organic residues in cementitious systems reports that publications have grown at approximately 30% per year, reflecting growing academic interest and industrial pressure [11]. A recent scoping review of agro-waste applications in structural building systems features coconut shell prominently among the candidate feedstocks, supporting the coconut-centered scope adopted here [3]. Within this review, 86% of the cited studies appear from 2022 onward and nearly half in 2025, and the cited work is grouped by theme, covering mechanical performance, durability and transport, microstructure, and sustainability, rather than by region or institution.

Three intervention routes recur throughout the literature: (i) organic aggregate substitution, with coconut shell as the primary candidate; (ii) lignocellulosic fiber reinforcement, including coir and other plant fibers; and (iii) agro-ash SCM blending. Smaller subsets of studies explore alkali-activated binders [7], with separate work applying machine-learning-based prediction or optimization to such mixtures [12].

A fourth route, combining coconut shell ash (CSA) with coir fiber in a single matrix, has also been reported; studies show that such hybrid systems show post-cracking benefit, though pore structure may be coarsened by CSA inclusion, with the net durability outcome depending on whether crack-bridging gains offset the porosity increase [13].

This review centers on coconut shell aggregate and coir/coconut fibers. Broader evidence from other lignocellulosic fibers and agro-ash SCMs is drawn on selectively where it sharpens the understanding of interfacial or pore-connectivity mechanisms, or supports hybrid-system reasoning, but the conclusions are not generalized beyond coconut-derived systems [5,7].

Beyond these core themes, a broad literature has examined coconut shell and related agricultural byproducts (e.g., date palm seeds) as partial coarse aggregate substitutes, coconut and coir fiber as crack-bridging reinforcement, and the durability, multifunctional, and secondary behaviors of the resulting composites; these studies are drawn on selectively in the relevant sections below rather than cataloged here [14,15,16,17,18,19,20,21,22,23,24,25,26,27,28,29,30].

Further studies extend this evidence base to the secondary and multifunctional behavior of coconut-based concretes, including the shrinkage behavior of coir-reinforced mixes under controlled environmental conditions [28], the strength and sound absorption of coconut fiber composites [29], and the thermal and radiation shielding performance of coconut shell ash blends [30].

Coconut-based materials have attracted considerable research attention, yet they remain largely absent from mainstream specifications. Part of the reason is that many studies report compressive-strength trends without also documenting stiffness, cracking behavior, or transport properties under relevant exposure conditions. In practice, these attributes, not strength alone, govern serviceability and durability, particularly in reinforced systems [31].

Variability is a second obstacle. Shell absorption and moisture conditioning both influence effective water content and ITZ quality; fiber geometry, surface treatment, and dispersion quality affect porosity and post-cracking behavior; and ash fineness and loss-on-ignition (LOI) affect water demand and admixture efficiency, with outcomes ranging from refined pore structure to increased porosity depending on how these variables interact [5,32].

These factors account for much of the apparent contradiction in the literature. Similar replacement ratios produce different outcomes not because of the ratio itself, but because of the full causal chain: replacement ratio, feedstock conditioning, effective water-to-binder ratio (w/b) control, consolidation quality, and curing regime. Tracking interfaces and pore connectivity mechanistically can reconcile many of these conflicting findings [5,7,31,33].

This review is organized around the interface-governed mechanisms (shell ITZ; fiber–matrix interphase) and pore connectivity that control transport, with explicit attention to the density–stiffness–transport trade-off in shell systems and the workability–porosity–toughness trade-off in fiber systems [31,32].

A key gap in the existing literature is the lack of a clear path from “material-level modification” to “application-ready mixture.” The paper addresses this by defining practical design windows, setting out what information is needed for conclusions to be reproducible, and grounding sustainability assessments in functional equivalency rather than per-volume comparisons [4,32].

Most studies remain at the material scale. Member-level studies exist but are limited, and exposure-class member durability is sparse. Structural interpretation is consequently treated as a matter of feasibility and translation, with conclusions bounded to avoid over-claiming [32,34,35].

**Figure 1:**
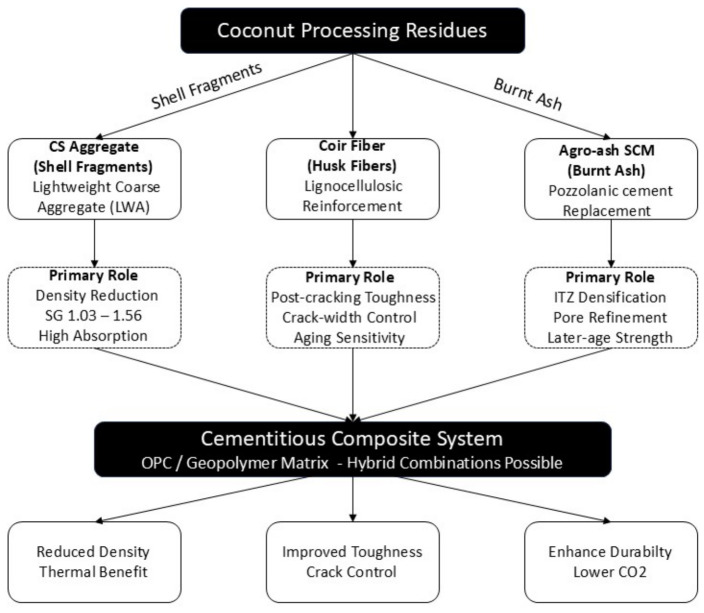
Three coconut-derived material routes in cementitious systems: CS aggregate (density reduction), coir fiber (post-cracking toughness), and agro-ash SCMs (ITZ densification and pore refinement).

This paper’s contributions are: (i) seven synthesis tables defining feasible design windows, performance ranges, and sustainability guidance; (ii) an account of interface and pore-connectivity mechanisms; (iii) a member-level translation synthesis; and (iv) functional-equivalency sustainability guidance [4]. The review draws on 98 peer-reviewed studies covering coconut shell aggregate, coir and coconut fiber reinforcement, and coconut-derived ash SCMs in cementitious systems, with selective reference to oil palm shell, jute fiber, and rice husk ash evidence where it sharpens mechanism interpretation. The three material routes and their respective roles are illustrated in Figure 1.

### Scope and Search Approach

This review is narrative and mechanism-focused rather than systematic, and reports no pooled meta-analysis. The cited literature spans 2014 to 2026. Studies were retrieved from Scopus and Web of Science, with Google Scholar used for supplementary coverage, using terms such as “coconut shell aggregate,” “coir/coconut fiber concrete,” “coconut shell ash,” “palm oil fuel ash” and “rice husk ash” combined with “mechanical,” “durability,” “ITZ” and “life-cycle”.

Peer-reviewed experimental and review articles reporting mix proportions and standardized test methods for coconut-derived constituents in cementitious systems were included; non-peer-reviewed material and studies without extractable mix or test detail were excluded. Primary experimental studies form the core evidence base, with reviews, life-cycle analyses, and analogous agro-waste studies used as supporting context. Because outcomes depend on feedstock conditioning and process control as much as on replacement ratio, the evidence is synthesized into bounded design windows).

Throughout, coconut shell aggregate and coir/coconut fiber studies form the core evidence base. The other materials and properties the review touches (palm oil fuel ash, rice husk ash, GGBS, steel fiber, oil palm shell, jute fiber, FRP, and the thermal, acoustic, and radiation shielding behavior) are treated as supporting evidence. They were included either because they share the mechanisms that govern coconut-based mixes (ITZ formation, pore connectivity, the fiber and matrix interphase) or because they bound the multifunctional performance of those mixes. Supporting studies are identified as such in the table footnotes.

Throughout, I separate what the literature reports from my own assessment of how far it can be trusted, and I state that assessment explicitly at the end of each section.

## 2. Materials, Processing Variability, and Minimum Reporting

Much of the scatter in reported outcomes traces back to variability in feedstock state and conditioning, not to the nominal replacement ratio. This is especially true for coconut shell (where absorption and moisture state are critical), coir fiber (where geometry and dispersion matter greatly), and agro-ash SCMs (where fineness and LOI strongly affect behavior) [5,32,36].

Coconut shell differs from conventional coarse aggregate in density, porosity, and absorption capacity. These properties enable density reduction, but they also make mixes more sensitive to water management and ITZ quality. When shell water demand is not properly controlled, the effective w/b ratio drifts from its target during mixing, leading to incomplete consolidation or unintended porosity. In many cases, these water-management effects dominate both strength and transport outcomes, obscuring the intrinsic contribution of the shell itself [5,9,37].

Studies across multiple source regions confirm that coconut shell (CS) aggregate specific gravity consistently falls in the range 1.03–1.56 and bulk density in the range 510–800 kg/m^3^, making it one of the lightest bio-aggregates available for structural concrete applications [1,31]. Aggregate size and fragment geometry also influence performance: larger fragment sizes tend to reduce abrasion value and improve particle interlock [38]. Quantitative ranges for all key CS aggregate properties, including comparison with other LWAs, are given in Table 1.

Practical mitigation strategies include controlled pre-saturation (e.g., saturated-surface-dry, SSD, conditioning), explicit accounting for water absorbed into the shell, and grading optimization to improve particle packing. Studies that document these controls consistently show more stable strength and durability data, with reduced batch-to-batch scatter [5,9,37].

### Feedstock Preparation and Modification

**Figure 2:**
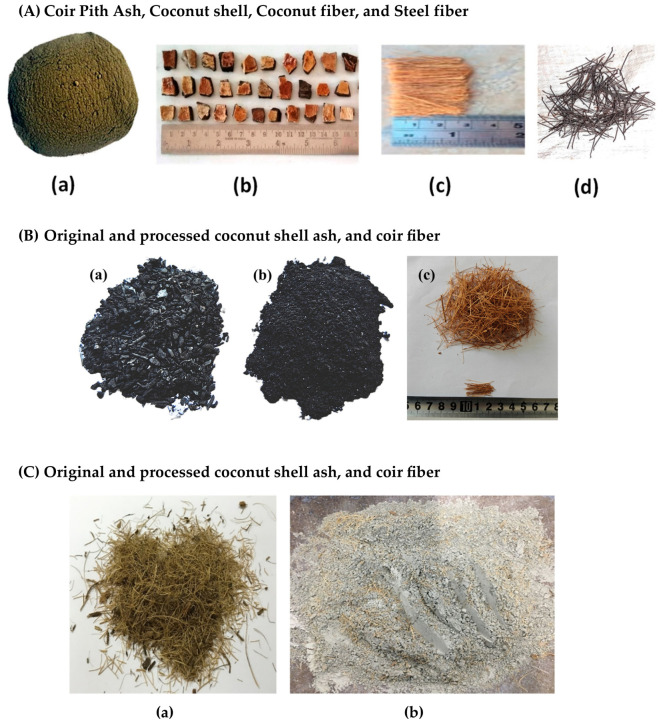
External appearance of the constituent materials: (**A**) Figure 1 of [5] showing (**a**) coir pith ash, (**b**) coconut shell aggregate, (**c**) coconut fiber, and (**d**) steel fiber; (**B**) Figure 2 of [13] showing (**a**) the original coconut shell ash (CSA), (**b**) processed CSA, and (**c**) coconut fiber (CF); (**C**) Figure 3 of [40] showing coconut fiber (**a**) ready for use and (**b**) in a dry mix.

The processing route is as decisive as the dosage. Coconut shell is crushed and graded (about 4.75–20 mm, nominal 12.5 mm) and conditioned to a defined moisture state before batching, since the saturated-surface-dry versus air-dried state of the shell governs the shell and matrix interface [5,31]. Coir/coconut fiber is cut to a controlled length (about 50 mm for high-strength mixes [40]) and may be surface-treated; citric acid treatment of coir lowers water absorption by 25–35% and sulfate-induced mass loss by 37–43% in geopolymer systems [7]. Coir pith ash and coconut shell ash are produced by controlled burning and calcination with fineness control, coconut shell ash being most effective near 5% replacement (a 27.65% compressive-strength increase) [4]. Reporting these steps is a pre-condition for reproducibility, since unrecorded conditioning is a primary source of the scatter discussed throughout the review. The external appearance of these constituent materials is shown in Figure 2.

Replacement of river sand with foundry sand alongside CS aggregate has been shown to improve packing density and form a dense matrix [46]. Substitution of sand with coir fiber combined with cattle manure has been investigated as a lightweight pairing, achieving approximately 50% reduction in thermal conductivity at 10% manure + 1.5% coir replacement [47]. Organic aggregate partial replacement studies confirm that cement composites can maintain acceptable strength when replacement is managed carefully and curing is controlled [9]. The use of coco-peat as a fine filler in foam concrete represents a further extension of the coconut material palette into novel applications [48].

When the shell is introduced dry, absorbed water reduces effective workability and can increase entrapped air. When the shell is pre-wetted without accounting, effective w/b can drift upward, increasing capillary porosity. These competing pathways explain why similar replacement ratios can produce divergent strength and transport outcomes across studies [5,9,37].

For context, recycled brick aggregate concrete combined with EPS beads has been investigated for masonry unit applications, showing comparable density ranges, with RBA exhibiting higher water absorption and lower density than natural aggregates [49]. Lightweight crumb rubber mortar provides a useful comparator for understanding workability trade-offs in low-density mixes [50].

**Figure 3:**
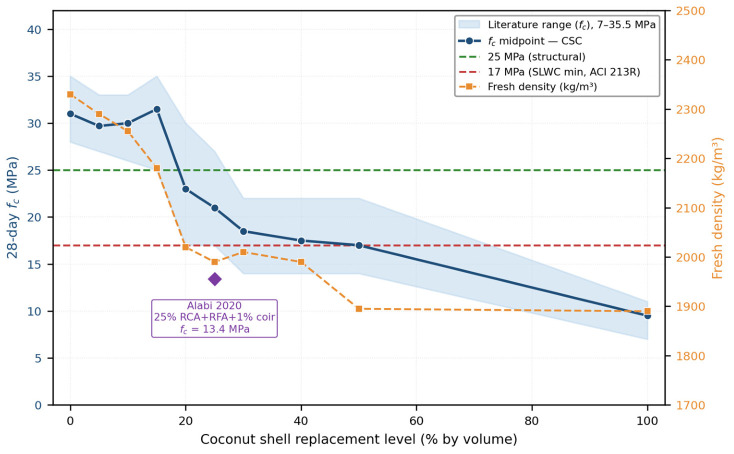
Twenty-eight-day compressive strength and fresh density of CSC vs. CS replacement level. Reference lines: 25 MPa (structural concrete) and 17 MPa (SLWC minimum, ACI 213R). Data from [9,31,51].

Replacement level is best discussed in bands rather than as a single optimum. The published data are too variable for a universal optimum to be meaningful; instead, Table 2 sets out low, moderate, and high replacement windows with the expected trade-offs in density, stiffness, serviceability, and transport risk [5,37]. The strength–replacement relationship is plotted in Figure 3.

Coir contributes primarily after cracking through bridging and pull-out, so tensile and flexural response, residual strength, and crack-width control are the outputs that actually matter for decision-making [10,52,53]. In contrast, compressive strength alone can understate fiber value or overstate fiber penalties when workability-induced porosity is not controlled [33,52,54]. Multi-strand coir rope configurations demonstrate tensile capacities exceeding 2 kN with progressive elongation response and no abrupt rupture, confirming the deformation-tolerant character that underpins toughness contributions at the composite level [41].

A clear separation must be drawn between short-term and long-term fiber performance. In the short term, moderate coir/coconut fiber dosages (optimum near 1–1.75% by binder mass) improve toughness, crack-width control, and residual capacity: a compressive peak of 55.1 MPa occurs at 1.75% fiber, about +24–26% above the unreinforced control (the source reports gains of 24% and 26% for compressive and axial-compressive strength) [40], and a silica fume and coir hybrid raises shear strength by about 70% at 1.5% coir with 5% silica fume [52]. Over the longer term, however, untreated natural fiber loses crack-control capacity under alkaline pore solution and wet–dry cycling. Accelerated weathering shows coir retaining about 95% of tensile strength at a three-year equivalent exposure but more than 70% loss by a five-year equivalent exposure [41]. Short-term toughness should therefore not be read as durable crack control. Surface treatment is one of the few measures shown to slow this loss. Citric acid modification of coir, for instance, lowers water absorption by 25–35% and sulfate-induced mass loss by 37–43% in geopolymer systems [7]. Table 3 consolidates the fiber design windows in Part A and the non-compressive performance indicators in Part B.

Agro-ash SCMs can refine pore structure through packing and pozzolanic effects and are frequently associated with later-age strength and reduced transport when ash quality and curing are controlled [5]. However, ash quality varies widely. High-LOI/high-carbon ashes can adsorb admixtures and increase water demand, worsening workability and increasing entrapped air; without adequate processing and mix adjustment, the intended refinement may not materialize [5,36].

Combined aggregate SCM use can reach structural-grade performance, the most mechanism-relevant result being that GGBS at an optimal 10% replacement in coconut shell concrete (CSC) raises compressive strength by about 15–20% and narrows the ITZ by 30–35% at 28 days, a direct densification pathway [31,44]. Other coconut-relevant SCM studies, used where they inform hybrid design, span self-compacting shell mixes with rice husk ash [55,56], biogenic silica from temperature-controlled ash [57], blended industrial and agricultural byproduct systems [58], biochar-based alkali–silica-reaction mitigation [59], coal-bottom-ash low-carbon mixes up to 66 MPa [60], and silica fume with coconut fibers [61].

In hybrid systems, SCMs often act as a “robustness layer” by refining the paste and ITZ and reducing connected porosity. This can partly offset shell-driven transport sensitivity and the porosity increases sometimes associated with fibers, provided that ash quality (e.g., reactivity) and curing are adequate [5,7,33].

Among the supporting agro-ash SCMs, palm oil fuel ash (POFA) is the most studied. Combined with metakaolin, it can exceed 75 MPa at optimized replacement [62]. In seawater sea sand concrete, it improves chloride resistance at about 10% replacement through pore refinement [45]. A critical review identifies fineness and LOI as its two primary quality variables [36]. Palm bunch ash [63] and palm-oil-clinker geopolymer aggregate, which reduces density from 2345 to 1821 kg/m^3^ with improved sulfate resistance [64], extend the same pozzolanic and packing mechanisms.

These supplementary cementitious materials are retained for scope-relevant reasons, not as independent topics. Rice husk ash, palm oil fuel ash, metakaolin, and GGBS share the mechanism that governs coconut-based mixes: as fine supplementary cementitious materials, they refine paste and ITZ porosity and offset the transport sensitivity that shell aggregate and high fiber dosages introduce. Coal bottom ash and geopolymer systems appear because the coconut-constituent studies relied on their use, namely the coir and coal-bottom-ash life-cycle case [60] and the citric acid coir durability data measured in a geopolymer matrix [7]. Coconut-derived ashes (coir pith ash, coconut shell ash) are themselves coconut feedstocks and are treated as core.

Much of the scatter between studies comes from how the materials are processed and reported, not only from the materials themselves. Shell moisture state, the basis used for fiber dosage (mass or volume), and ash quality (fineness, loss on ignition) all shift the outcome, yet they are seldom reported. Pooling results into a single optimum would therefore be misleading, so this review gives bounded design windows rather than single values and states which reporting gaps would need to be closed before a quantitative meta-analysis could be justified. The mechanics-of-materials literature provides established methods for tracing this kind of input variability through to the scatter in measured properties [65].

In my view, a central reporting weakness in this field is that moisture conditioning and ash quality are treated as incidental details instead of as primary independent variables. Until they are reported as routinely as the replacement ratio, much of the apparent disagreement between studies will be hard to attribute to the materials themselves rather than to how they were prepared and reported.

## 3. Mix Design and Constructability

Fresh-state behavior is the first gate that determines both mechanical and durability outcomes. Many of the performance penalties reported in agro-waste concrete studies stem not from intrinsic material incompatibility, but from poor water balance control, inadequate fiber dispersion, or excessive entrapped air [33,52,54].

In shell-containing mixes, the effective w/b is governed by shell absorption, pre-saturation state, and mixing sequence. Adding dry shell to the mix draws water out of the paste, reducing slump and risking incomplete consolidation. Conversely, pre-wetting without careful control can push effective w/b upward and inflate capillary porosity. Both pathways affect strength and transport, making moisture conditioning a variable that must be controlled and documented, not left as an afterthought [5,9,37].

Shrinkage behavior is also altered in organic aggregate mixes: the lower stiffness and higher absorption of bio-aggregates modify paste–aggregate stress transfer, and early-age shrinkage can be elevated if moisture management is not controlled [66].

For fiber mixes, placement and dispersion often control the outcome more than the nominal dosage. As fiber surface area increases, flow can collapse, and fiber networks can trigger balling, segregation, and air entrainment; the threshold depends on aspect ratio, dosage basis, mixing energy, and admixtures [33,52,54]. For that reason, studies that report and control workability and dispersion are more interpretable than those that report only hardened results [33,52,54]. In hybrid coir–steel systems, slump reductions as high as 66.67% have been reported when fiber combinations are not carefully managed [67].

Agro-ash SCMs modify paste demand and rheology through fineness and carbon content. Fine ashes can improve packing, but high-LOI ashes can adsorb superplasticizers, reducing their effectiveness and forcing water adjustments. In hybrid systems, these effects compound: shell absorption changes the effective water content, fibers reduce flow, and fine ashes increase paste demand. If these interactions are not managed, increased air content and poor consolidation dominate both strength and transport response. This mechanism explains why some hybrids show improvement while others show mixed results despite similar nominal replacement ratios [5,7,33]. Without these descriptors, conclusions about constituent effects on durability remain confounded by consolidation artifacts [31,54].

In practical quality control terms, shell and fiber systems behave as “water-and-air sensitive.” In such systems, fresh-state acceptance criteria (minimum flow/slump and maximum air content, plus qualitative dispersion checks) can be as important as compressive strength for ensuring reproducible performance. This perspective aligns with the mechanism by which pore connectivity governs transport and durability [31].

The most consistent approach in shell mixtures is to pre-condition the aggregate to a defined moisture state, similar to SSD conditioning used for porous lightweight aggregates [9].

Mixing sequence matters because it determines when and where water is absorbed. A frequent source of scatter is adding shell late into the mix (after paste formation) without accounting for rapid uptake, which can increase local paste viscosity and promote incomplete coating and higher void content. Conversely, a pre-wetted shell added with controlled surface moisture tends to reduce these artifacts [5].

Rheology in fiber mixes depends heavily on how fibers are introduced. Pre-soaking can reduce water competition but can also introduce free water if not controlled; surface treatments can improve interphase but may alter wetting behavior and dispersion. These interactions explain why the treatment protocol and mixing sequence need to be documented alongside mix proportions, not treated as secondary details [10,32,33].

Pumpability and surface finishing are both sensitive to shell angularity and fiber networks. Though most studies omit pumping or placement data, constructability discussions grounded in fresh-state mechanisms (such as water balance, entrapped air, and segregation resistance) strengthen practical relevance even when field trials are scarce [31]. Hybrid mixtures, in particular, demand a carefully considered water budget alongside a deliberate rheological strategy.

Beyond laboratory batching, constructability is often the decisive filter for whether agro-waste modifications can be used in real projects.

In hybrid mixtures, interactions are not simply additive. Shell increases water demand and can increase ITZ sensitivity; fibers increase surface area and can lock the mixture; fine SCMs may increase paste demand and change admixture efficiency (especially when LOI is high). In a well-constructed hybrid, water budget and rheology are the primary design variables; strength and durability outcomes follow from whether pore connectivity was tightened or worsened [5,7,31,33].

Controlling the fresh state of a hybrid mix rests on three steps. First, pre-condition the shell aggregate to a saturated-surface-dry state, or apply a measured pre-soak water correction, so that aggregate absorption does not drive progressive slump loss during mixing and placing [5,31,37]. Second, meet the extra water demand from coir/coconut fiber and from fine supplementary cementitious materials with a water-reducing or polycarboxylate superplasticizer, dosed by trial batch to a target slump rather than by adding water, which would raise the effective water-to-binder ratio and cancel the SCM benefit [5,16,52]. Third, keep fiber at or below its optimum dosage and disperse it well, since high or poorly dispersed fiber both stiffens the mix and entrains air; air content should be checked on trial batches because both fiber and angular shell tend to raise it [9,52,67]. Because these effects are mix-specific, admixture type and dosage are best fixed by trial batching for the particular shell grading, fiber length, and SCM combination.

My position is that constructability should be treated as a first-order performance criterion for these materials instead of a practical afterthought. A hybrid mix that cannot be placed and consolidated at controlled air content has already surrendered the durability advantage its constituents were selected to deliver.

## 4. Mechanical Response and Serviceability Implications

Compressive strength alone is insufficient for interpreting mechanical performance. Stiffness, crack initiation behavior, and post-cracking response frequently govern serviceability limits and long-term durability, particularly in reinforced concrete elements [31].

Studies on natural fiber RC systems confirm that toughness, crack initiation, and ductility/energy absorption are the outputs that distinguish fiber-reinforced from plain concrete [35,42,68,69]. Hybrid natural–synthetic fiber combinations in recycled aggregate concrete show that synergistic effects on toughness and durability can be achieved when fiber types are matched to failure mode [70]. The addition of steel fibers to CS concrete has been shown to enhance flexural strength and ductility, with 10% fly ash as a partial cement replacement further improving the overall response [35]. Coconut fiber dosage optimization confirms that the peak response for most mechanical indicators falls in the 1–1.75% range by mass of binder [13,33,40,42,52,71].

Two points need to be kept separate when interpreting this literature: (i) compressive-strength retention is not the same as structural feasibility, and (ii) early-age trends do not guarantee durable performance. Durability is largely governed by connected porosity and crack state, so strength should be read together with stiffness and the intended exposure [31]. Table 4 consequently emphasizes stiffness implications rather than strength trends alone [5,37].

Strength trends across shell studies are mixed because they depend on conditioning, packing, and curing. Optimized grading and moisture conditioning can stabilize workability and reduce unintended porosity, which tends to reduce scatter and sometimes recover strength at moderate replacement levels. Conversely, high replacement with uncontrolled absorption can induce porosity and reduce both strength and durability proxies. Replacement ratio alone is not a reliable predictor; conclusions should be conditioned on whether effective w/b control and consolidation quality were actually demonstrated [5,9,37]. A broad review of coconut shell ash systems reports optimum compressive strength increments of 27.65% at 5% CSA replacement, with flexural and tensile strength increments of 52.24% and 28.54% respectively, at their respective optimum dosages, and density ranges of 2349–2514 kg/m^3^ consistent with light-to-normal weight concrete production [4]. The compiled strength–replacement relationship across the reviewed studies is shown in Figure 3.

**Figure 4:**
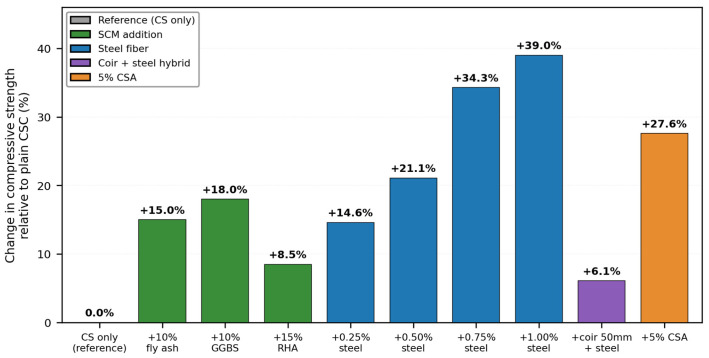
Effect of SCM and fiber additions on CSC compressive strength relative to plain CSC. Low-level SCM additions improve strength; steel fiber at 0.75–1.0% produces the largest gains. Data from [3,4,15,31,35,67,72,73].

In contrast, SCM refinement and adequate curing can densify paste and ITZ, reducing transport and sometimes improving later-age mechanical stability. Where such refinement is absent, durability risks associated with absorption and transport can increase even if compressive-strength targets are met [32]. The quantitative effect of SCM and fiber additions on compressive strength is summarized in Table 4 (Part A) and illustrated in Figure 4.

Oil palm shell (OPS) is a closely related lightweight aggregate and a useful comparator: it has been investigated as a structural lightweight aggregate, including in fiber-reinforced members under impact loading [27], and geopolymer OPS concrete has been characterized under elevated temperature [74].

Fiber systems (toughness–workability–aging): Coir fibers contribute primarily to post-cracking response through bridging and pull-out, so the decision-relevant outputs are residual flexural/tensile capacity, toughness indices, and crack-width control rather than compressive strength alone [5,10,52]. Under controlled dispersion, moderate fiber dosages can improve ductility and energy absorption with a limited compressive strength penalty. At higher dosages, workability loss and air entrainment can dominate, producing apparent strength losses that are partly processing artifacts [33,52,54].

A consistent pattern runs through these studies: workability falls with fiber content (jute behaves much like coir [75]), and peak compressive gains for coconut fiber occur near 1–1.5% [3,42,76,77]. For coconut shell coarse aggregate replacement, the optimum is about 10% with bending strength losses of 15–41% between 15% and 30% replacement [78], while using coir as a sand replacement cuts compressive strength by 32–83% over 25–100% replacement, placing its useful range below about 25% [79]. Coconut fiber alone gives only modest compressive benefit and can underperform plain concrete [3,76]; combining waste streams, however, remains viable (1% coir with 25% recycled aggregate reaches 13.4 MPa [51]). Coir–steel hybridization recovers the coir penalty, with compressive and splitting tensile gains of 6.13% and 8.42% at 50 mm and 75 mm fiber length [67].

Long-term retention is a critical constraint for exposure-class claims. Lignocellulosic fibers are sensitive to alkaline pore solution and moisture cycling, which can alter interphase properties and pull-out behavior over time. Short-term toughness gains should not, on their own, be read as evidence of durable crack control. Where aging evidence is available, it should be treated as a boundary condition on claims [32,33].

The influence of natural cellulosic fiber on concrete water absorption depends critically on fiber type and loading percentage [69].

**Figure 5:**
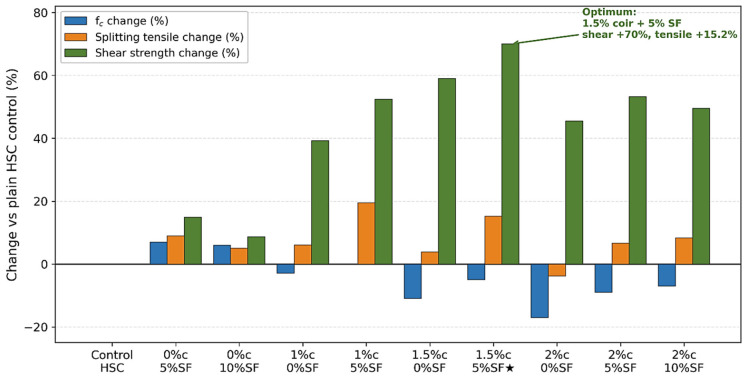
Changes in compressive, splitting tensile, and shear strength for coir fiber and silica fume combinations in HSC (fc > 42 MPa). The star (★) on the x-axis marks the optimum mix (1.5% coir + 5% SF), which gives shear strength +70%. Data from [52]; values at 28 days.

Hybrids can be strong when each component has a clear role: shell for density reduction, fibers for crack control, and SCMs for pore refinement and later-age stabilization. Hybrids are also more sensitive to QC because they combine multiple water-demand and rheology modifiers. Coir fiber and silica fume interaction data in high-strength concrete are tabulated in Table 4 (Part B), and the resulting mechanical response is illustrated in Figure 5.

Normalization to internal controls at the same age and curing improves interpretability across binder systems. Reporting variability (replicate count, scatter) is also important because high scatter is itself an adoption barrier for natural-constituent composites [31].

Mechanical synthesis should be read alongside durability. Strength results divorced from transport proxies and exposure context are difficult to interpret in practice. This integrated view aligns with functional-equivalency screening [31].

**Figure 6:**
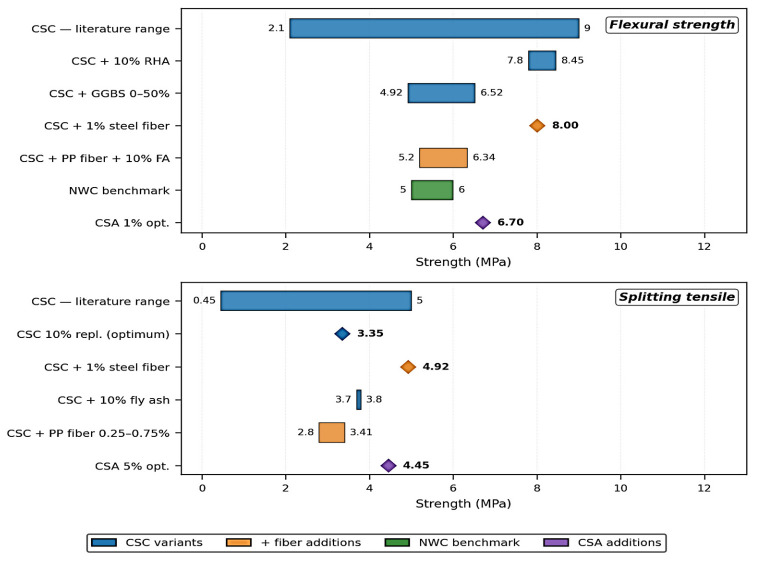
Flexural (**upper group**) and splitting tensile (**lower group**) strength ranges for CSC and coir-reinforced systems. Steel fiber addition shifts CSC flexural strength to or above the NWC benchmark. Data from [4,31,73].

Across coir and other lignocellulosic fibers, the main structural contribution is improved post-cracking response (toughness/residual capacity). Most studies report gains at modest dosages, while higher dosages frequently reduce workability and can increase porosity. The practical optimum is therefore mix- and process-dependent rather than universal [5,10,33,52,54,80]. Figure 6 collates reported ranges for flexural and splitting tensile strength.

A broad review of fiber types confirms that fiber-reinforced concrete benefits in toughness and crack control are consistent across natural fibers, with durability governed by interphase stability [81,82]. Experimental data from coconut fiber concrete studies confirm that 1.5% fiber content by weight of binder consistently produces the largest post-cracking toughness gains [42].

Treating fiber as an energy-dissipating, crack-bridging phase aligns with broader work on dissipation in enriched cementitious systems [83].

Hybrid systems can be positioned as risk-managed designs: SCM refinement can strengthen the matrix and densify the ITZ, supporting both shell and fiber performance. Where ash quality and curing are controlled, later-age performance and transport can improve relative to shell-only mixes; where controls are absent, hybrids may simply compound fresh-state sensitivity [5,7,33].

For fiber mixtures, crack-width control matters only if it remains stable under aging. The evidence is easiest to interpret when short-term crack-control results are separated from results after alkaline/wet–dry exposure, and when transport testing is performed on cracked specimens [32,33].

Damage-softening constitutive models for palm fiber concrete demonstrate that post-peak behavior can be captured analytically when fiber geometry and volume fraction are explicitly parameterized [84]. Mechanical behavior studies on palm fiber concrete provide additional evidence that natural fiber concretes follow predictable constitutive trends when dispersion is controlled [85].

What counts as a structural-grade claim needs to be defined clearly. For fiber systems, that means post-cracking indicators and crack-width control, not compressive strength alone [5].

When shell concretes are proposed for structural use, member-scale evaluation under realistic service loads should be part of the evidence [31].

**Figure 7:**
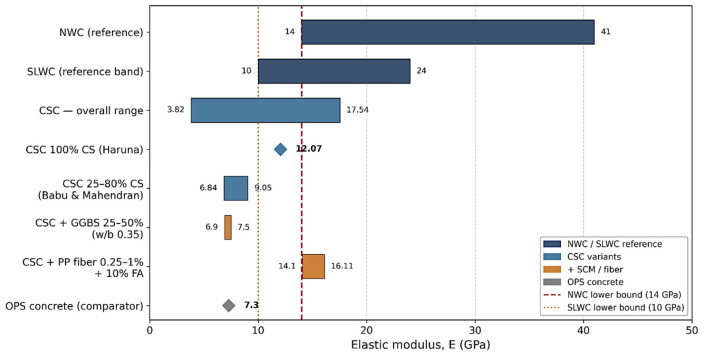
Elastic modulus ranges for CSC variants relative to NWC and SLWC reference bands. Most CSC systems fall well below the NWC lower bound (14 GPa, dashed line). Data from [31].

Quantitative synthesis can be strengthened by reporting typical ranges of modulus reduction and noting that SCM refinement may partially recover stiffness through a denser ITZ, even when pooled statistics are not feasible [7,10]. The elastic modulus ranges for CSC variants relative to NWC and SLWC reference bands are shown in Figure 7.

In my view, the field’s reliance on compressive strength as the headline metric is one of the main barriers to credible structural claims for these systems. For fiber concretes in particular, a result reporting compressive strength alone tells us little about the post-cracking behavior the fiber was added to provide, which is why I give more weight to residual capacity and crack-width control when judging whether a study supports a structural claim.

## 5. Microstructure Property Mechanisms

Many apparent contradictions in this literature become explicable when viewed through a mechanism-centered lens. The controlling variables are the quality of interfaces and the degree of connected porosity. The interfaces that matter most are the ITZ around shell particles and the fiber–matrix interphase. SCM addition and adequate curing can densify both paste and ITZ, improving stiffness and reducing transport; but fresh-state entrapped air and consolidation defects can negate these benefits entirely [7,10,31,32].

In shell concretes, the ITZ is the critical microstructural zone. The porosity and surface texture of shell particles influence local packing and hydration dynamics around each particle, and a more porous or mechanically compliant ITZ simultaneously reduces composite stiffness and opens preferential pathways for moisture and ion transport. This explains why modulus reduction and increased absorption so often occur together at higher replacement levels. When SCM addition and adequate curing are used to refine the matrix, the observed improvements in transport and mechanical stability are attributable primarily to densified ITZ and reduced connected porosity, not to any change in the shell itself [7,10].

In fiber systems, the fiber–matrix interphase governs load transfer and pull-out friction. Treatments may improve initial bond, but alkaline pore solution and moisture cycling can change the interphase over time. Interphase characterization is of limited value unless it is paired with aging or durability testing [10,32,33].

Chemical treatment of coconut fibers (including alkaline and silica fume surface modification) has been shown to improve fiber–matrix adhesion and enhance mechanical performance [43]. In corroded reinforced self-healing concrete, coconut fiber has been shown to slow crack propagation and support partial self-healing mechanisms, highlighting the durability dimension of fiber–matrix interphase quality [86]. Constructability is not just a practical issue; it directly shapes durability because dispersion and entrapped air control connected porosity and transport [5,7,31,33].

One additional mechanism relevant across systems is shrinkage-related microcracking. Changes in paste content, water balance, and fiber networks can alter shrinkage behavior. Microcracking increases connected porosity and accelerates ingress, coupling shrinkage and cracking indicators to durability, as shown in Table 5 and reinforcing the link between serviceability and durability [33].

For strong interpretation, microstructure evidence is most valuable when tied to decisions: reduce replacement, adjust conditioning, improve dispersion, or incorporate refinement strategies. The review therefore emphasizes causal chains that link processing → interface/pore structure → mechanical/durability response [7,31,33].

**Figure 8:**
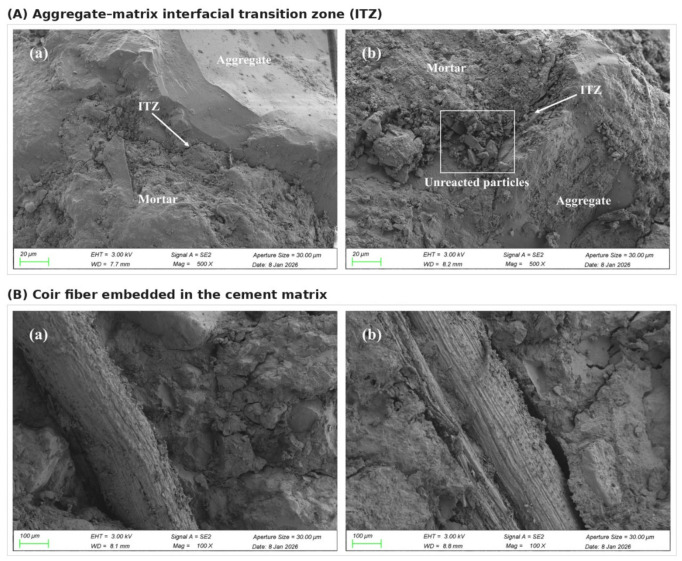
Composite SEM micrographs of coconut-derived cementitious systems, reproduced from [13]: (**A**) aggregate–matrix interfacial transition zone (ITZ), showing (**a**) the ITZ between aggregate and mortar in the control mix, and (**b**) the ITZ with coconut shell ash, where unreacted particles are visible within the interfacial region (scale bars: 20 µm); (**B**) coir fiber embedded in the cement matrix, showing (**a**) overview of the fiber–matrix interaction, and (**b**) close-up of the fiber surface and matrix contact (scale bars: 100 µm).

Microstructural evidence supports the proposed mechanisms: image analysis of the interfacial transition zone shows GGBS narrowing the ITZ by about 30–35% at 28 days [44], while the rough, convex outer face of crushed coconut shell improves the shell–matrix bond [31]. Figure 8 illustrates these microstructural features at both the aggregate–matrix ITZ and the coir fiber–matrix interfaces.

In my assessment, the weak point of this evidence is that connected porosity, the variable this section identifies as controlling transport, is almost never measured directly; it is inferred from strength or absorption rather than from pore-network characterization. Until pore connectivity is measured and tied to a transport result, the processing-to-property chain stays partly assumed rather than demonstrated.

## 6. Durability/Transport and Thermal Performance

Durability claims need to be grounded in two realities: the conditioning history of the specimens and their crack state at the time of testing. Shell-containing mixes frequently show elevated absorption at higher replacement levels unless matrix refinement is applied; for fiber systems, durability depends on whether crack-control benefits persist under aging [31,32,33,37].

Durability synthesis should distinguish between (a) matrix transport in an uncracked state and (b) ingress controlled by cracking and microcracking under service loads. This gap explains why many structural-durability claims remain conditional: not because the effect is unknown in principle, but because the testing configuration often does not reflect the governing service state [32,33].

For shell concretes, increases in absorption and sorptivity at higher replacement are consistent with porous aggregate pathways and ITZ sensitivity. These indicators do not automatically disqualify the material, but they imply that exposure class and protective strategies matter. When SCM refinement is used, multiple studies report reduced transport proxies relative to shell-only mixes, consistent with pore refinement [7,31,37].

Coconut shell aggregate size variation has a measurable effect on concrete durability: water absorption decreases as shell fragment size increases, so smaller fragments raise absorption at equivalent replacement levels [38,87]. Permeability and dry shrinkage in oil palm shell concrete follow comparable trends, with surface treatment of OPS particles reducing both sorptivity and shrinkage relative to untreated controls [88].

Chloride-related proxies (diffusion/rapid chloride permeability test, RCPT) are more directly relevant for corrosion risk than absorption alone. Credible durability claims should name both the test and conditioning and avoid equating unrelated indices [32].

Aging of natural fibers remains a central limitation for long-term durability interpretation. Long-term retention of crack control is the key missing link between short-term toughness gains and service-life claims, especially under wet–dry cycling and alkaline exposure [32,33].

Thermal performance trends are often more consistent than durability: lower-density mixes generally show lower thermal conductivity, supporting blocks and panels. Elevated temperature residual strength is protocol-dependent (heating rate, heating duration, matrix composition), so synthesis should summarize it as regime-dependent rather than as a single ranking [79,89,90].

Thermal conductivity of coconut shell concrete decreases with increasing shell replacement, with values in the range 0.4–0.8 W/mK reported across 10–100% replacement; these reductions track density reduction rather than replacement level per se [91]. Elevated temperature exposure modifies microstructure and pore size distribution in sustainable concrete, with the degree of damage depending on moisture content at the time of heating and the heating rate [90]. Bio-fiber-based roofing and panel systems exploit the low thermal conductivity of natural fiber-reinforced matrices to achieve insulation performance comparable to commercial fiber and gypsum boards [92,93,94]. Porous coconut shell concrete also demonstrates improved sound absorption coefficients relative to conventional concrete, opening applications in acoustic panels [3,31]. Coconut shell concrete with coconut shell ash incorporation shows promising gamma radiation shielding characteristics, with the linear attenuation coefficient responsive to ash content and barite modification [72,95]. Palm microfibers in concrete improve thermal performance, with fiber addition reducing thermal conductivity by 26–33% on average at optimum dosage [73]. A structured summary of what is consistently reported versus what remains conditional in the thermal and high-temperature domain is given in Table 6.

A common weakness in this literature is claiming “improved durability” from a single short-term indicator without documenting specimen conditioning or crack state. Credible durability claims must identify the governing mechanism, whether pore refinement or crack control, report conditioning and crack state explicitly, and link findings to an exposure-relevant index for reinforced applications [31].

Absorption and sorptivity are useful screening metrics, but their meaning depends on conditioning, curing history and crack state. Without these controls, identical mixes can appear more or less durable due to conditioning differences [31,32].

**Figure 9:**
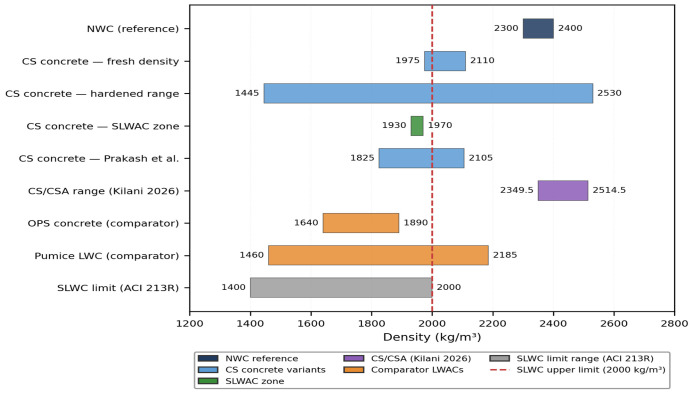
Density ranges for CSC variants vs. NWC and other LWACs. Dashed line: SLWC upper limit (2000 kg/m^3^, ACI 213R). Most CSC systems sit at or below this boundary. Data from [4,31,73].

Shell concretes present a characteristic durability risk profile: absorption and sorptivity tend to increase with higher replacement because shell porosity and ITZ effects increase connected pathways. The density ranges for CSC variants relative to NWC and other LWACs, which underpin this risk profile, are shown in Figure 9. This does not mean shell concretes are unusable; it means durability must be treated as a design constraint that can be mitigated by matrix refinement, curing control, and, in some cases, surface protection. The evidence supports conditional feasibility rather than unconditional improvement [5,37].

For reinforced applications, chloride transport proxies (diffusion preferred; RCPT was used) are more directly relevant than absorption alone. Where available, results suggest matrix refinement can reduce transport, whereas porous aggregates and poor consolidation can increase it. Conclusions should therefore be bound by binder chemistry, curing regime and crack state [32].

### Consolidated Quantitative Entries (Durability and Transport, Splitting Tensile, and Chloride Proxy)

(i) Citric acid surface treatment of coir and jute fibers lowers water absorption by 25–35% and sulfate-induced mass loss by 37–43% in geopolymer systems [7]; (ii) under accelerated weathering, coir retains about 95% of tensile strength at a three-year-equivalent exposure but loses more than 70% by a five-year-equivalent exposure [41]; (iii) GGBS narrows the coconut shell concrete interfacial transition zone by about 30–35% at 28 days, the parameter that governs transport [44]. Each value is taken directly from the cited study. Splitting tensile strength follows the same dosage logic: it rises 28.54% at 5% coconut shell ash [4]; a coir and silica fume hybrid gives the largest gain at 1.5% coir with 5% silica fume, with smaller gains of about 4–6% at 1–1.5% coir alone and a decline at 2% [52]; and coconut fiber in a geopolymer matrix peaks near 0.5%, falling 4.64% at 1% [53]. For chloride, the cited studies report the rapid chloride permeability proxy (charge passed) rather than a true diffusion coefficient: 20% coconut shell ash lowers the total charge passed by 35.3% [13], and coir pith ash mixes are reported as low chloride-ion penetrability under ASTM C1202 [5]. True chloride diffusion coefficients are seldom reported across these studies, one of the reporting gaps this review identifies.

For fiber systems, the durability benefit is expected primarily through crack-width control. However, a credible claim requires evidence that crack control persists under aging, and that transport under the cracked state is improved or not worsened. The scarcity of such combined evidence is one of the more consequential gaps in the literature [32,33]. The full set of relevant durability and transport indicators, together with interpretation notes, is provided in Table 5.

Which durability tests are relevant depends on the application. Absorption/sorptivity screen moisture sensitivity, chloride diffusion/RCPT proxies relate to corrosion risk, sulfate resistance indicators matter in sulfate-bearing soils, and carbonation indicators matter for cover depth and atmospheric exposure. Mapping test-to-exposure relevance adds value beyond listing outcomes [7,32,33].

Another issue is that durability indices can be influenced by cracking induced during mechanical testing, see Table 5.

Sustainability synthesis is strongest when it separates potential from verified under functional equivalency. Many papers report per-m^3^ embodied CO_2_ reductions, but functional equivalency requires comparable strength and durability class (or service life). Without this, a lower-carbon mixture that performs poorly in transport or requires thicker sections may not reduce life-cycle impact [4,32].

In the reviewed literature, clinker substitution via SCMs is consistently positioned as the most direct CO_2_-reduction pathway, whereas the benefit of aggregate substitution depends strongly on transport distance and processing. Sustainability conclusions should include sensitivity to local supply chains rather than making universal statements [4,7,32].

This aligns with Table 7 and reduces over-claiming without adding unnecessary complexity [4,32].

Durability is a sustainability variable. Even without full service-life modeling, explicitly stating that durability screening is part of environmental evaluation strengthens credibility because premature deterioration increases life-cycle impacts through repair and replacement [4,32].

Thermal conductivity generally decreases as density decreases, which makes coconut shell systems attractive for lightweight blocks and panel applications. Residual strength after heating, however, depends strongly on the test regime (initial moisture condition, heating rate, and matrix/binder composition). For that reason, elevated temperature results should only be compared across studies with similar conditioning and thermal protocols, and any conclusions should report the regime rather than implying universal fire-performance gains [89].

Based on the available evidence, a practical durability qualification sequence is to: (i) verify fresh-state quality to minimize porosity-related artifacts; (ii) assess absorption and sorptivity under standardized preconditioning; (iii) include a chloride-related indicator for any reinforced application; and (iv) interpret results in light of binder chemistry, curing regime, and specimen crack state.

My reading of the durability evidence is that it tends to run optimistic, because the dominant tests are short-term, uncracked, and single-property, and so rarely reproduce the governing service state, while aged and cracked-state transport data remain scarce. I therefore treat reported durability benefits for fiber systems as provisional until they are shown to persist under aging and in the cracked state.

## 7. Structural Translation and Member-Level Evidence

Material-level data alone cannot justify the structural use of agro-waste concretes. Member response depends on stiffness, cracking behavior, reinforcement interaction, boundary conditions, and loading type. Material-scale tests do not capture these variables [31,35]. Member-level studies in the reviewed literature offer valuable feasibility evidence, but they remain sparse compared to the wealth of material-scale data, and exposure-class durability at the member scale is particularly limited [34,35].

Member-level evidence is best treated as translation rather than definitive code-level validation. The strongest member studies report both load capacity and serviceability (deflection, crack development), because serviceability is where modulus differences and crack-control mechanisms most clearly emerge [34,35].

**Figure 10:**
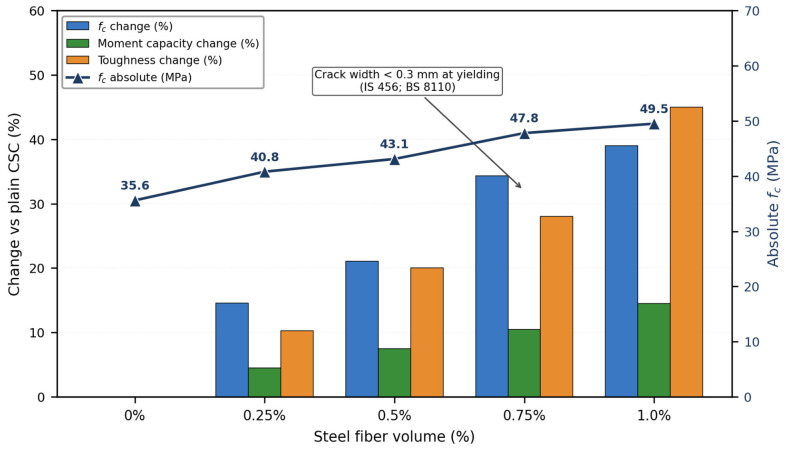
Effect of steel fiber content on CSC mechanical performance at member scale; crack widths at yielding remain below 0.3 mm in SCS-CSC sandwich beams (IS 456:2000; BS 8110). Data from [3,8,34,35].

Natural hybrid FRP-strengthened RC beams demonstrate that member-level flexural performance can be maintained or improved relative to conventional concrete beams when fiber dosage and composite action are optimized [96]. Structural performance assessments of lightweight fiber-reinforced RC members confirm that serviceability limits (deflection and crack width) are more sensitive to stiffness reduction than ultimate capacity, reinforcing the need for modulus reporting alongside strength [35,96]. The effect of steel fiber addition on CSC compressive strength, moment capacity, and toughness at the member scale is summarized in Figure 10.

Member evidence should therefore be interpreted together with modulus and exposure demand, especially for reinforced elements in chloride environments [31].

In fiber systems, translation depends on whether fibers produce stable crack-width control under relevant loading and environmental conditioning. Member studies can demonstrate mechanical feasibility, but durability translation still requires cracked-state ingress evidence [32,97].

A conservative, application-oriented summary is that near-term adoption is most defensible in non-structural components and moderately exposed elements where density/thermal benefits matter, while aggressive exposure structural applications require stronger transport and serviceability evidence [3,31,37,78].

The distinction between structural and material evidence matters here. Member-scale results indicate feasibility for specific configurations, not blanket suitability across exposures and structural systems [31,35].

For fiber concretes, crack-control benefits must persist under aging to translate into durability improvement [31,32].

Where member-scale feasibility evidence exists (e.g., composite systems, confinement or hybrid reinforcement contexts), it is useful primarily as translation guidance. Such studies should report serviceability indicators (deflection, crack width) alongside ultimate capacity, because these serviceability indicators are often more sensitive to modulus differences than ultimate strength [34,35].

Member results should be interpreted with attention to boundary conditions and composite action assumptions. This is another reason why a review should not generalize from one member type to all structural forms [34,35].

The evidence points toward near-term adoption being most defensible for non-structural or moderately exposed applications such as blocks, panels, and partition elements, where density reduction and thermal performance are the primary objectives and transport screening can be performed. Structural applications are technically plausible, but claims should be explicitly conditional, requiring demonstrated modulus adequacy, serviceability screening, and exposure-relevant transport testing such as diffusion or RCPT [3,31,37,78].

My position is that the present evidence supports these materials for non-structural and moderately exposed components, while general structural use is better treated as conditional than assumed. The member-scale studies are useful but configuration-specific, and in my assessment, the evidence that would most change this decision is cracked-state, exposure-relevant testing at the member scale, which remains among the scarcest data in the field.

## 8. Sustainability and Functional Equivalency

Environmental benefits only materialize when a mix also meets performance and durability requirements. If it does not, design compensation such as thicker sections, additional reinforcement, or more frequent maintenance can erode or entirely negate the embodied-impact savings; this is evident when carbon is normalized per unit strength, where embodied-carbon-per-MPa can worsen as strength falls [32,79]. The sustainability evidence across the reviewed studies is synthesized under a functional-equivalency framework in Table 7, and the practical reporting checklist for sustainability comparisons is provided in Table 7 (Part B).

Sustainability comparisons are only meaningful under functional equivalency: first confirm feasibility (strength class, serviceability demand, and exposure-relevant durability), then compare embodied impacts among mixes that deliver the same function using transparent boundaries and allocation assumptions [4,32].

Sustainability synthesis benefits from being explicit about what is compared. Functional equivalency means comparing mixes that deliver the same structural function under similar durability demand. If a shell mixture reduces modulus, a structure may require more material or reinforcement, changing embodied impact at the component level. Per-m^3^ comparisons only mean something after feasibility has been confirmed [4,32].

Within this framing, clinker substitution by SCMs is the most direct pathway to embodied-CO_2_ reduction, but the ranking depends on boundary and allocation assumptions. Many studies use different allocation choices for waste materials; strong synthesis should state that allocation can change conclusions and recommend sensitivity analysis [4,32].

Aggregate substitution benefits depend strongly on transport distances and processing energy. In some contexts, local sourcing makes bio-aggregates attractive; in others, transport or processing can erode benefits. Claims should be tied to regional supply-chain conditions and avoid blanket statements such as “always lower carbon” [4,32]. Incorporating recycled aggregates alongside coir fiber in OPC concrete directly addresses both waste streams simultaneously, with combined RCA, RFA and 1% coir fiber mixes achieving compressive strength up to 13.4 MPa in controlled laboratory conditions [51]. Hybrid coir–steel fiber systems similarly advance sustainability objectives by extending the performance envelope of low-dosage natural fiber additions without requiring high cement contents [67]. Broad reviews of coconut shell and shell ash systems confirm that 0–5% CSA substitution for cement can increase compressive strength beyond that of control concrete while reducing binder consumption, offering a co-benefit of environmental and mechanical performance [4].

Life-cycle assessment studies comparing natural, recycled, and conventional concrete confirm that the environmental advantage of bio-aggregates is most pronounced when transport distances are minimized and local processing infrastructure is available [98]. Hybrid machine learning models combining LCA data with strength predictions demonstrate that the optimal sustainable mix depends simultaneously on compressive strength, transport distance, and processing energy, with no single dominant variable [12]. Broader reviews of coconut and palm kernel shell systems conclude that incorporating CSA and PKSA in cement and concrete addresses CO_2_ emission reduction, environmental waste management, and concrete sustainability simultaneously, with these materials suitable for light-to-normal-weight green concrete production [4].

Embodied-carbon accounting (total CO_2_ per cubic meter, as the sum over constituents of mass times CO_2_ coefficient) shows cement dominating the footprint. Cradle-to-gate inventories place Portland cement at about 0.90–0.96 kg CO_2_/kg (0.93 [8]; 0.898 [32]; 0.96 [60]), with water at zero, and aggregates and supplementary cementitious materials an order of magnitude lower (fine aggregate about 0.0139 and coarse aggregate about 0.0408 [8]; silica fume about 0.0011 [32]; coal bottom ash about 0.05 [60]). Natural fiber is low but source-dependent (coir 0.20 [8] versus 0.003 [60]; jute 0.36 [32]). Reductions therefore come mainly from displacing clinker: a coir and coal-bottom-ash concrete has been reported to lower embodied carbon by 194 kg CO_2_/m^3^ against its control, and embodied energy from 2101 to 970 MJ/m^3^ [60]. Because the decision-relevant quantity is carbon per unit of delivered performance, a strength-to-embodied-carbon metric (MPa per kg CO_2_/m^3^) rises from 0.1269 for the plain Portland-cement control to 0.357 for a coir and coal-bottom-ash mix, a 179.3% gain [60], while conventional concrete sits near the low end at 0.072 [8]. Blanket low-carbon claims are therefore avoided, and conclusions are framed per unit of delivered strength [8,32,60].

Durability is a sustainability variable, not a separate consideration. A modification that increases permeability and shortens service life may well increase life-cycle impact even if it reduces cradle-to-gate CO_2_. This linkage is increasingly recognized in recent studies; the practical approach is to reach sustainability conclusions only after durability qualification, and to state explicitly where service-life data are missing [4,32].

A straightforward way to communicate sustainability results is a screen-and-rank approach: (i) screen by performance and durability (strength class, modulus/serviceability, transport indicators), (ii) among feasible mixes, compare embodied impacts under stated assumptions, and (iii) report sensitivity to transport distance and allocation. This workflow aligns with Table 7 and helps prevent over-claiming [4,32].

Because coconut residues are geographically concentrated, transport can dominate the result. LCA reporting should explicitly state transport distances and the electricity mix used for processing; otherwise, the conclusions are not transferable across regions [4,32].

My view is that the sustainability case for these materials still rests on relatively few life-cycle studies that compare mixes on a common functional unit. The functional unit comparisons that do exist, such as the strength to carbon gain reported for a coir and coal-bottom-ash mix [60], are encouraging but isolated, and they rarely pair the carbon figure with a durability or service-life estimate. Until that pairing becomes routine, I regard a net carbon advantage for bio-based mixes as plausible but not yet established.

## 9. Research Roadmap and Conclusions

The evidence base is uneven, and this review separates established findings from emerging ones. Material-scale strength, density, and modulus trends for coconut shell concrete are reasonably well supported, whereas member-scale structural behavior and long-term in-service durability rest on far fewer studies, with long-term experimental datasets especially scarce. These are treated as conditional: the performance ranges given here are indicative design windows rather than predictive guarantees, and durability and structural feasibility.

The positions below are my own judgment of the evidence, not a neutral summary.

What this review adds is a mechanism-based reading that ties processing inputs to interface and pore-structure outcomes: bounded design windows in place of single optima, an explicit separation of core coconut evidence from supporting agro-waste evidence, and a functional-equivalency basis for sustainability comparison.

Key takeaways

- Coconut shell aggregate can reduce density, but stiffness/serviceability and moisture-driven transport sensitivity are recurrent constraints unless grading, moisture conditioning, and matrix refinement are controlled.
- Coir/coconut fibers primarily improve post-cracking response and crack-width control when dispersion is maintained; long-term interphase stability under alkaline and wet–dry exposure remains the dominant uncertainty for exposure-class claims.
- Agro-ash SCMs can densify pore structure and reduce transport where ash quality (fineness/LOI) and curing are adequate; otherwise, increased water demand and variability can offset benefits.
- Sustainability comparisons are most defensible under functional equivalency: screen mixes by performance, serviceability, and exposure-relevant durability before comparing embodied impacts.

Main evidence gaps

- Limited cracked-state transport datasets for fiber-modified systems under controlled crack widths.
- Sparse long-duration aging datasets linking fiber interphase evolution to retained crack control and transport.
- Member-scale validation under exposure-relevant durability protocols remains limited compared with material-scale testing.

Priority future work

- Standardize reporting of shell moisture conditioning, fiber dosage basis, and ash quality indices (fineness/LOI) to reduce false disagreements across studies.
- Adopt combined protocols that couple controlled cracking with transport measurement and aging exposures for fiber systems.
- Develop integrated material–member datasets where the same mix is evaluated across fresh state, modulus/toughness, transport proxies, and member response.
- Beyond the reporting items noted above, studies should report a minimum set of mix proportions and at least one mechanical and one transport property under stated curing and exposure, so that results become directly comparable. Because outcomes depend strongly on local feedstock, grading, and exposure, interlaboratory comparison on common reference mixes would do most to establish which reported benefits are reproducible rather than local.

Practical recommendations

- Assign each constituent a single role: shell for density reduction, fiber for crack control, and supplementary cementitious material for pore refinement. Keep coir/coconut fiber near its optimum dosage (about 1.75%). Pre-condition the shell aggregate to a defined moisture state before batching. Use the supplementary cementitious material (for example, coconut shell ash at a rate of 5%) to offset the porosity introduced by shell and fiber.

## Figures and Tables

**Table 1:** Coconut shell aggregate properties and comparison with other LWAs.

**Part A. Coconut Shell Aggregate Properties**				
Parameter	Typical Reported Range	Implication for Mix Design		Key Sources
Bulk density (kg/m^3^)	≈510–800	Lightweight organic aggregate; varies by source and grading.		[1,31]
Specific gravity (−)	1.03–1.56	Lower than natural aggregates; contributes to density reduction.		[1,31]
Water absorption (%)	≈14–29	High absorption drives effective w/b sensitivity; conditioning required.		[5,9,37]
Particle size/grading	Study-specific	Replacement level should be interpreted together with grading/PSD.		[10,39]
**Part B. Comparison with other lightweight aggregates**				
Aggregate	Specific gravity (–)	Bulk density (kg/m^3^)	Water absorption (%)	Note
Coconut shell (CS)	1.03–1.56	510–800	13.79–29.11	47–60% lighter than granite NWA
Oil palm shell (OPS)	1.17–1.35	530–685	18.69–33.0	Natural LWA from oil-palm processing residues
Pumice	0.99–2.84	395–1305	2.59–60.3	Natural volcanic LWA
Scoria	1.31–2.68	710	2.00–19.20	Natural volcanic LWA
Perlite	0.12–0.73	111–200	63–188.10	Expanded volcanic glass
Granite (NWA)	2.30–2.97	1491–1682	0.40–1.50	Conventional coarse aggregate
**Part C. Summarizes the preparation, treatment, and quantitative characterization of the constituents**				
Constituent	Preparation procedure	Treatment/modification	Quantitative characterization	Key sources
Coconut shell aggregate	Crushed and graded ~4.75–20 mm (nominal 12.5 mm); conditioned to a defined moisture state before batching	SSD pre-saturation or measured pre-soak water correction; grading optimized for particle packing	Specific gravity 1.03–1.56; bulk density 510–800 kg/m^3^; water absorption ~14–29%; ~47–60% lighter than granite NWA	[1,5,9,31,37,38]
Coir/coconut fiber	Cut to controlled length (~25–75 mm; ~50 mm common and often optimal for high-strength mixes); dosed by mass or volume with dispersion control	Surface modification: alkaline, silica fume, or citric-acid treatment	Citric-acid treatment lowers water absorption 25–35% and sulfate-induced mass loss 37–43% (geopolymer); accelerated weathering ~95% tensile retention at 3-yr-equivalent, >70% loss by 5-yr-equivalent	[7,40,41,42,43]
Coir pith ash/coconut shell ash (CSA)	Controlled burning and calcination with fineness control	Replacement-level optimization (most effective near 5%)	At ~5% CSA: +27.65% compressive, +52.24% flexural, +28.54% splitting tensile (respective optima); 20% CSA lowers RCPT charge passed by 35.3%	[4,13]
Supporting SCMs (GGBS, POFA, RHA)	Used as fine partial cement replacement; quality set by fineness and loss-on-ignition (LOI)	Replacement-level optimization (commonly ~10%)	GGBS ~10% in CSC: +15–20% compressive, ITZ narrowed 30–35% at 28 d; POFA ~10% improves chloride resistance; high LOI raises water demand	[31,36,44,45]

**Table 2:** Mechanical performance data and decision windows for coconut shell concrete.

**Part A. Mechanical Performance Data by Replacement Level (fc, Density, E)**				
CS Replacement (vol.%)	fc, 28 d (MPa)	Density (kg/m^3^)	E (GPa)	Key Observation
0% (NWC control)	~28–34	2300–2400	14–41	Reference: conventional concrete
≤10–15% (low)	25–35	2035–2165	10–17	Strength is often maintained; some studies show marginal gains at 5–10%
~15–30% (moderate)	17–28	1930–2100	7–15	Density target met; serviceability screening required
≥30–50% (high)	9–20	1445–1975	4–10	Non-structural use more appropriate; large E reduction
100% CS	9.29	~1880–1930	3.82–7	Density qualifies as structural lightweight concrete (SLWC); structural use requires mitigation
**Part B. Decision-oriented replacement windows and expected trade-offs (range-based synthesis)**				
Replacement window	Density/strength trend	Serviceability/stiffness implication	Durability/transport implication	Key sources
Low (≤10–15% vol.)	Small density reduction; fc often maintained	Modulus penalty may be moderate; screen E and cracking	Transport risk is manageable with curing/SCM	[10,37,39]
Moderate (≈15–30% vol.)	Clear density reduction; fc may decline unless optimized	Serviceability often governs due to the E reduction	Combine with SCM pore refinement and conditioning	[5,37]
High (≥30–50% vol.)	Strong density reduction; fc variability increases	Large E reduction is likely; non-structural is more realistic	Higher absorption/transport risk unless mitigated	[5,37]

Replacement basis: by volume. Test age: 28 days. Sources are cited per window in the table. Water-to-binder ratio and shell conditioning for each window: Low window [10,37,39]: water-to-binder is kept low and paired with a plasticizer, with the shell used saturated-surface-dry [37] or pre-soaked to a saturated state before batching [39]; the cited review summarizes conditioning practice rather than one mix [10]. Moderate and high windows [5,37]: water-to-binder near 0.33 [5], shell again conditioned saturated-surface-dry [37]. Where a study did not state an exact ratio or a conditioning step, the entry is marked not reported. Note: the replacement windows in Part B are indicative ranges synthesized from heterogeneous studies; outcomes remain conditional on shell grading, moisture conditioning, effective w/b control, and curing/test protocol [10,31].

**Table 3:** Coir/coconut fiber design windows, mechanisms, and practical constraints (range-based synthesis).

**Part A. Design Windows, Mechanisms, and Practical Constraints**			
Design Variable	Typical Reported Range	Mechanistic/Engineering Implication	Key Sources
Fiber length (mm)	25–75 (common); 50 often “best” in factorial studies	Controls pull-out length and bridging efficiency.	[42]
Fiber content	≈0.25–2% (mass) or low vol.% ranges	Higher contents reduce workability; risk of balling/air.	[33,42,52,54]
Treatment	Alkali/surface treatment (study-specific)	Often improves interphase and residual capacity; report protocol.	[7,10,32,52]
Primary benefits	Toughness, crack control, residual strength	Benefits strongest when dispersion is controlled.	[10,52]
Primary risks	Workability loss, porosity increase, aging	Need cracked-state durability + aging evidence.	[32,33]
**Part B. Non-compressive performance indicators**			
Design variable	Typical reported range	Mechanistic/engineering implication	Key sources
Shear capacity (post-crack)	+~70% at 1.5% coir + 5% silica fume	Post-crack shear resistance.	[52]
Splitting tensile	~+47% at 1.5% coir + 5% silica fume; only ~4–6% with coir alone	Tensile/bridging gain at the optimum.	[52]
Strain/energy absorption	Coir sustains 4–6× the strain of other natural fibers	Energy-absorption capacity.	[42]
Residual/post-crack capacity	Improved, concentrated near the 1.75% optimum	Durable crack-control window.	[40]
Toughness, deflection/ductility	Reported as load-deflection/shear curves, not discrete indices; “Not reported” marks indicators that a source did not quantify.	Reporting gap to standardize.	[42,52]

Note: The design windows in Table 3 summarize typical ranges reported in the literature and should be used as decision aids rather than prescriptive standards; dispersion quality, mixing energy, and curing strongly influence outcomes [33,52,54].

**Table 4:** Effect of SCM and fiber additions on coconut shell concrete mechanical performance [31,35,52].

**Part A. Effect of SCM and Fiber Additions on CSC Mechanical Performance**				
SCM Addition	fc Change	E Change	Flexural Change	Source
10% fly ash	+15–16%	Partial recovery	Improved	[31]
10% GGBS	+15–20%	Partial recovery	Improved	[31]
25% GGBS	−8% to −14%	Reduced	−18%	[31]
50% GGBS	−22% to −26%	Reduced	−18%	[31]
15% RHA	+8.5% vs. control	Not reported	+3.0%	[31]
0.75% steel fiber + CS	+34% vs. CS alone (47.8 vs. 35.6 MPa)	Increased	+45% (toughness, [30]); up to +52% (strength, [4])	[4,31,35]
0.25–1% steel fiber + CSC	+15–39% vs. CS alone (40.8–49.5 vs. 35.6 MPa)	Increased	+~14% moment capacity	[3,31,35]
**Part B. Coir fiber and silica fume interactions in HSC (Ali et al. 2022 [52])**				
Coir/SF combination	Compressive change	Splitting tensile change	Shear change	Note
1% coir, 0% SF	−4%	+6%	+39.3%	Compressive penalty small; shear gain is significant
1.5% coir, 0% SF	−15%	+4%	+59%	Workability limit approaching
1.5% coir, 5% SF	−15% + SF gain	Highest tensile	+70%	Optimal combination for HSC
2% coir, 0% SF	−17%	Negative	+45.5%	Workability loss dominates; porosity increases
0% coir, 10% SF	+16%	+minor	Minor	SF alone: pore refinement effect

Changes reported relative to CS concrete control at equivalent replacement level. Fly ash and low-level GGBS additions show consistent strength and transport improvement. High GGBS levels reduce strength, suggesting an optimal SCM window. Steel fiber at ≥0.5% volume fraction produces consistent moment capacity and toughness gains at member scale [3,35]. Data from 50 mm long coir at specified % by weight of binder, in HSC with 28-day fc > 42 MPa. Shear strength was measured on 150 × 150 × 150 mm specimens. At 1.5% coir with 5% SF, the shear strength of HSC increased by more than 70% relative to plain control [52].

**Table 5:** Durability/transport indicators and interpretation notes for coconut shell and coir systems.

Metric/Domain	Typical Direction Across Studies	Interpretation Notes	Key Sources
Absorption/sorptivity	Often higher for shell mixes at higher replacement	Report conditioning & crack state; mitigate via SCM/cure	[4,37]
Chloride transport proxy (RCPT/diffusion)	Often improved by SCM refinement; can worsen with porous fibers or aggregates	Prefer diffusion; interpret with curing	[44,52]
Sulfate/chemical attack indicators	Mixed; depends on pore refinement and protocol	Report concentration/duration; normalize to control	[7]
Shrinkage/cracking indicators	Fibers can reduce cracking but may alter shrinkage trends	Crack-state governs ingress; report crack width	[28,33]
Aging of fibers	Alkaline + wet–dry cycling can reduce interphase performance	Long-term validation still limited	[32,33]

**Table 6:** Thermal and high-temperature performance: what is consistently reported versus conditional.

Domain	Typical Reported Trend	Interpretation Notes	Key Sources
Thermal conductivity	Typically decreases with density reduction	Useful for blocks/panels/insulation products	[3,91]
Elevated temperature residual strength	System-dependent; sensitive to moisture & heating regime	Avoid generalization; report protocol	[89]
Special functions (acoustic/radiation)	Reported in a subset of studies	Application-specific; requires validation	[29,72,95]

**Table 7:** Sustainability synthesis and reporting checklist.

**Part A. Sustainability Synthesis Under Functional Equivalency**			
Item	What the Literature Reports	Rationale	Key Sources
Functional unit	Often volume-based; best practice includes performance/exposure class	Avoid per m^3^ comparisons across unequal strength/service life	[13,30]
Clinker substitution	Most direct embodied-CO_2_ reduction pathway	Depends on ash quality and replacement level	[7,13]
Aggregate substitution	Can reduce virgin aggregate demand	Net benefit depends on transport + serviceability compensation	[4,32]
Allocation method	Varies widely (including zero-burden assumptions)	Must be stated; run sensitivity	[30]
Durability linkage	Rarely quantified; often assumed	Do not claim CO_2_ benefit if durability is compromised	[33,64]
**Part B. Functional-equivalency reporting checklist. Note: values are indicative ranges synthesized from heterogeneous studies [31]**			
Item	What to report	Rationale	
Functional unit	1 m^3^ meeting strength & exposure class (or equivalent structural function)	Avoids unequal comparisons across strength classes	
System boundary	Cradle-to-gate/site; include processing/transport as relevant	Boundary choice can change the ranking	
Waste allocation	Mass/energy/economic/zero-burden; justify choice	Allocation can reorder options	
Performance constraints	Strength + modulus/serviceability + durability thresholds	Sustainability must not ignore durability	
Assumptions & sensitivity	Transport distances, electricity mix, curing sensitivity	Tests the robustness of conclusions	

## Data Availability

No new data were created or analyzed in this study. Data sharing is not applicable to this article.
